# Time Course of Wrist Hyper-Resistance in Relation to Upper Limb Motor Recovery Early Post Stroke

**DOI:** 10.1177/1545968320932135

**Published:** 2020-06-07

**Authors:** Aukje Andringa, Carel Meskers, Ingrid van de Port, Erwin van Wegen, Gert Kwakkel

**Affiliations:** 1 Department of Rehabilitation Medicine, Amsterdam UMC, Vrije Universiteit Amsterdam, Amsterdam, The Netherlands; 2 Department of Physical Therapy and Human Movement Sciences, Northwestern University, Chicago, IL, USA; 3 Revant Rehabilitation Center, Breda, The Netherlands; 4 Department of Neurorehabilitation, Amsterdam Rehabilitation Research Centre, Reade, Amsterdam, The Netherlands

**Keywords:** stroke, muscle spasticity, upper extremity, biomechanical phenomena, recovery of function

## Abstract

*Background*. Patients with an upper limb motor impairment are likely to develop wrist hyper-resistance during the first months post stroke. The time course of wrist hyper-resistance in terms of neural and biomechanical components, and their interaction with motor recovery, is poorly understood. *Objective*. To investigate the time course of neural and biomechanical components of wrist hyper-resistance in relation to upper limb motor recovery in the first 6 months post stroke. *Methods*. Neural (NC), biomechanical elastic (EC), and viscous (VC) components of wrist hyper-resistance (NeuroFlexor device), and upper limb motor recovery (Fugl-Meyer upper extremity scale [FM-UE]), were assessed in 17 patients within 3 weeks and at 5, 12, and 26 weeks post stroke. Patients were stratified according to the presence of voluntary finger extension (VFE) at baseline. Time course of wrist hyper-resistance components and assumed interaction effects were analyzed using linear mixed models. *Results*. On average, patients without VFE at baseline (n = 8) showed a significant increase in NC, EC, and VC, and an increase in FM-UE from 13 to 26 points within the first 6 months post stroke. A significant increase in NC within 5 weeks preceded a significant increase in EC between weeks 12 and 26. Patients with VFE at baseline (n = 9) showed, on average, no significant increase in components from baseline to 6 months whereas FM-UE scores improved from 38 to 60 points. *Conclusion*. Our findings suggest that the development of neural and biomechanical wrist hyper-resistance components in patients with severe baseline motor deficits is determined by lack of spontaneous neurobiological recovery early post stroke.

## Introduction

Recovery of post-stroke upper limb motor impairment is heterogeneous. Recent studies suggest that most patients follow a predictable pattern of spontaneous neurobiological recovery within the first 3 months after stroke, while 20% to 30% of the patients fail to show any motor recovery.^1-3^ Previous observational studies have shown that early control of voluntary finger extension (VFE) is an important determinant of upper limb motor recovery at 6 months post stroke.^4,5^ In addition, several studies suggested that patients with poor motor recovery are likely to show increased resistance to passive muscle stretch,^6-8^ that is, hyper-resistance. This hyper-resistance is hypothesized to be caused by a poorly understood and complex interaction between pathological neuromuscular activation due to damage to descending pathways as well as non-neural biomechanical changes in the muscles and soft tissues spanning the joint post stroke.^9-11^ The neural components of hyper-resistance may be divided into velocity-dependent stretch hyperreflexia (altered set point and/or gain of the stretch reflex, ie, spasticity following the definition of Lance)^9,12^ and non-velocity-dependent involuntary activation (ie, increased background levels of contraction).^11,13^ Biomechanical components of joint hyper-resistance include altered tissue properties, for example, elasticity, viscosity and muscle shortening.^11,14^

In particular, there is a lack of knowledge about the time course of wrist hyper-resistance in terms of its neural and biomechanical components, and its interaction with motor recovery early post stroke,^15^ yet this is important for understanding observed improvements in motor control of the upper paretic limb in terms of behavioral restitution and compensation strategies.^16,17^ Development of the velocity and non-velocity-dependent neural components, among which spasticity, as a reflection of reorganization of spared descending pathways, might reflect neural repair processes during upper limb recovery, further influencing behavioral restitution.^16,18^ Moreover, information about the time course of different components of wrist hyper-resistance may help to optimize individualized treatment decisions, for example, when and to whom to apply botulinum toxin treatment^19-21^ during the early post-stroke phase. Considering the target mechanism of botulinum toxin, blocking neural signal transmission to the muscle, it is expected that patients with an increased neural component of wrist hyper-resistance will benefit most from this treatment. Recently, a new measurement technique, called NeuroFlexor (Aggero MedTech, AB), has been developed for the quantification of neural and biomechanical elastic and viscous components of wrist hyper-resistance, which has proved to be valid^22,23^ and reliable^23,24^ in patients with chronic stroke.

The first aim of the present study was to investigate the time course of wrist hyper-resistance in the first 6 months post stroke, separated into its neural and biomechanical elastic and viscous components. This was done in patients with and without VFE within 3 weeks post stroke, in relation to the critical time-window of spontaneous neurobiological recovery as reflected by improvements observed using the Fugl-Meyer upper extremity scale (FM-UE). Findings were compared with healthy reference data. The second aim was to investigate the association between neural and biomechanical elastic and viscous components of wrist hyper-resistance in the first 6 months post stroke.

We hypothesized that in patients without VFE within 3 weeks post stroke, in the absence of spontaneous neurobiological recovery, both the neural and biomechanical components of wrist hyper-resistance would gradually increase over time.^15^ In addition, we hypothesized that the neural component would increase within the time-window of spontaneous neurobiological recovery, while an increase of the biomechanical components would not be restricted to this specific time-window. In a similar vein, we hypothesized that an increase in the neural component would be accompanied by an increase in biomechanical components, in reaction to a pathological neuromuscular activation. In patients with VFE within 3 weeks post stroke, that is, those showing spontaneous neurobiological recovery, components of wrist hyper-resistance were hypothesized to normalize to values seen in age- and gender-matched healthy subjects.

## Methods

### Participants

All patients with stroke who were admitted to Revant Rehabilitation Center Breda, The Netherlands, for inpatient rehabilitation were screened for eligibility between July 2015 and July 2016. The inclusion criteria for this study were (1) a first-ever ischemic stroke within the past 3 weeks, with an initial upper limb deficit as defined by the National Institutes of Health Stroke Scale item 5 a/b score >0 (ie, not able to hold the affected arm at a 90° angle for at least 10 seconds), (2) ≥18 years of age, (3) able to sit in a chair for at least 1 hour, and (4) sufficient cognitive ability to follow test instructions as indicated by a score higher than 17 on the Mini Mental State Examination.^25^ Exclusion criteria were (1) a history of other neurological impairments and (2) limitations of arm-hand function of the affected side prior to the stroke. A group of healthy, right-handed, age- and gender-matched adults without wrist function restrictions served as a reference group. Ethical approval was obtained from the Medical Ethics Reviewing Committee of the VU University medical center, Amsterdam, The Netherlands (protocol number 2014.140). In accordance with the Declaration of Helsinki (2013), all participants gave written informed consent.

### Study Design and Procedures

In this prospective cohort study, repeated measurements were performed at fixed times post stroke, that is within 3 weeks, and at 5, 12, and 26 weeks. The first measurement was performed as soon as possible after stroke onset, with more intensive repeated measurements within the window of nonlinear spontaneous neurobiological recovery within the first 12 weeks post stroke^3^ and a follow-up measurement at the start of the chronic phase after stroke.^26^ Demographics and stroke characteristics were collected at baseline. All measurements were performed by a trained assessor. In the healthy controls, neural and biomechanical components of wrist hyper-resistance were determined for the dominant arm. All patients received usual care. The use of botulinum toxin injections was recorded throughout the study period.

At baseline, patients were stratified into 2 groups, based on the presence or absence of VFE within 3 weeks post stroke^4,5^: (1) a group of patients showing any VFE, according to the FM-UE item of finger extension >0, within 3 weeks and (2) a group of patients showing no VFE (FM-UE item finger extension = 0) within 3 weeks.

### Outcome Measures

**Figure 1.**
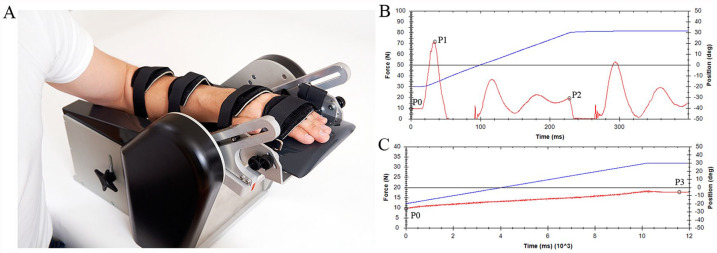
NeuroFlexor method. (A) Measurement set-up. (B) An example of the force trace (red line) obtained during a fast movement (236°/s). (C) An example of the force trace obtained during a slow movement (5 deg/s). The blue line represents the angle of the wrist joint. The recorded force traces, measured in newton (N), are analyzed by a biomechanical model, which results in the quantification of the velocity-dependent part of the neural component (NC), elastic component (EC), and viscous component (VC) of wrist hyper-resistance. The total measured resisting force (F_m_) during passive wrist extension is a summation of passive elastic force (F_p_), viscous force (F_v_), reflexive force (F_r_), and inertial forces of the limb and the moving parts of the device (F_in_), described as: F_m_(θ) = F_p_(θ) + F_v_(θ) + F_r_(θ) + F_in_(θ), where θ denotes a specific angle. In the model, 4 force points in the resistance trace of the slow and fast movements are used to estimate the different components of the total measured passive force. P0 is the resting torque of the hand before onset of stretch. Two force points are defined within the fast passive wrist extension movement (236 deg/s): P1, the initial peak in resistance, and P2, the late peak in resistance. One force point (P3) is defined at the end position of the slow wrist extension movement (5 deg/s). Resting torque (P0) is subtracted from P1, P2, and P3 prior to further calculations. Detailed information about the biomechanical model can be found in Supplemental File 1.

Neural and biomechanical elastic and viscous components of resistance to passive wrist extension were assessed with a validated and commercially available measurement technique, the NeuroFlexor, feasible for use in clinical practice (Figure 1).^22^ This motor-driven device imposes isokinetic wrist displacements with extended fingers from 20° palmar flexion to 30° dorsal flexion at 2 controlled velocities (5 and 236 deg/s), for which a minimal passive wrist extension of 40° is needed. A force sensor, placed underneath the moveable hand platform, measures the resistance trace during the passive wrist movement. The participant was seated comfortably parallel to the device with the shoulder in 45° of abduction, 0° of flexion, the elbow in 90° of flexion, with the forearm fastened to the device in pronation, and the hand with extended fingers fastened to the hand platform. Participants were instructed to relax their arm and to look ahead of them during the measurements. The experimental session consisted of 5 slow movements (5 deg/s) followed by 10 fast movements (236 deg/s). The first movement at both velocities was excluded from analysis to avoid bias from startle reflexes and mechanical hysteresis. The resting torque of the hand before onset of stretch was subtracted from the resistance traces prior to further calculations. Using the biomechanical model described by Lindberg et al,^22^ the different components of wrist hyper-resistance, that is, the velocity-dependent part of the neural component (NC), the biomechanical elastic component (EC), and viscous component (VC), were derived from the resistance traces (using the NeuroFlexor Scientific v0.06 software program, Supplemental File 1 and Supplemental Figure 1). The NC was determined as a derivative of the velocity-dependent resistance to passive wrist extension, which is to reflect the neural, velocity-dependent part of wrist hyper-resistance, that is, assumed proxy of spasticity as defined by Lance,^12^ not including the non-velocity-dependent part of neural activity, that is, involuntary background activation. The length-dependent EC was determined as the resistance at the end of the slow movement. It was assumed that the velocity-dependent VC was highest during the initial acceleration and continued at a lower level, that is, 20%, during further extension movement. The developers of the NeuroFlexor have previously underpinned the validity of the NC based on 3 arguments: (1) the NC as measured by the device was reduced after an ischemic nerve block, (2) the NC correlated with the integrated electromyography (EMG) across subjects and in the same subject during the ischemic nerve block, and (3) the NC was found to be velocity dependent.^22^ In a recent study,^23^ the NeuroFlexor method was suggested to be construct-valid against clinical assessments using the modified Ashworth and Tardieu scales. In addition, good to excellent reliability was shown for the quantification of the different components.^23,24^ As a result of the positioning of the fingers, the measured resistance was a combination of resistance caused by wrist and finger flexor muscle groups. Measurements were performed twice at the same occasion, and mean values were used for further analysis.

Synergy-dependent motor recovery of the upper limb, as a reflection of spontaneous neurobiological recovery, was assessed by the FM-UE^27^ with a scoring range from 0 to 66 points. To test voluntary finger extension, patients were instructed to open the hand as much as possible starting from the resting position of the wrist and fingers, and the forearm in a neutral position between pronation and supination (0 = no voluntary extension movement in the metacarpophalangeal (MCP) or interphalangeal (IP) joints occurs, 1 = any degree of extension movement in the MCP or IP in any finger and/or thumb, 2 = full extension movement of all fingers that is equal to or greater than the unaffected side). Good measurement properties of the FM-UE have been established in studies of patients with stroke.^28,29^

Total resistance to passive wrist extension with extended fingers was measured manually using the modified Ashworth scale (MAS),^30^ an ordinal scale with scores ranging from 0, no increased tone, to 4, the joint is rigid.

### Statistical Analysis

All analyses were performed using IBM SPSS Statistics for Windows, version 22.0 (IBM Corp). Descriptive statistics were used for demographic and clinical characteristics.

Linear mixed model analyses were used to investigate the time course of neural and biomechanical components of wrist hyper-resistance during the first 6 months post stroke, and the interaction between this time course and the prognosis of upper limb motor recovery as defined by presence or absence of VFE within 3 weeks post stroke. Fixed effects were modeled for the time and group factors, and for the interaction between time and group. To correct for dependencies between the measurements, a random intercept per participant was used. For all 3 components, assumptions of normally distributed residuals were confirmed by inspecting histograms and Q-Q plots. Statistical level of significance was set at .050.

Statistical analysis of the difference in neural and biomechanical components between patients and healthy controls was performed using the Mann-Whitney U test. The associations between FM-UE and NC and EC at week 26, as well as between neural and biomechanical components over time, were calculated using Pearson correlation coefficients. Correlation coefficients below 0.25 were classified as no to little, 0.25 to 0.50 as fair, 0.50 to 0.75 as moderate to good, and greater than 0.75 as good to excellent association.^31^

## Results

**Figure 2.**
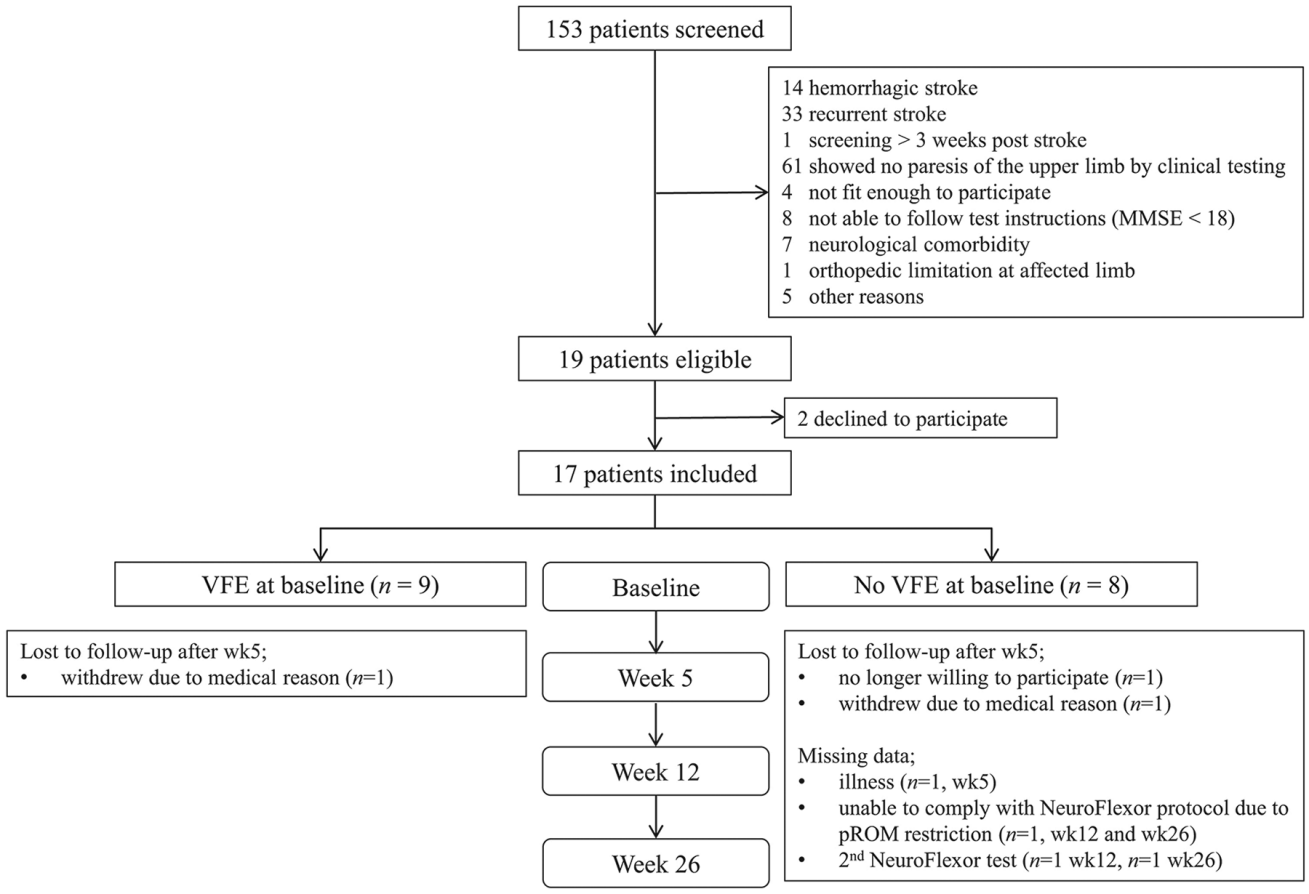
Flowchart.

Figure 2 shows the participant flowchart. A total of 153 patients were screened for eligibility, and 17 were included within 3 weeks post stroke. The demographic and clinical characteristics of the patients at baseline are summarized in Table 1. The baseline measurement was performed on average 15 ± 4 days post stroke (range 8-19 days). At baseline, 9 patients showed any VFE while 8 patients showed no VFE. Seventeen age- and gender-matched healthy controls (11 males and 6 females, mean age 60 ± 8 years, all right-handed) were included in the study.

**Table 1.** Demographic and Clinical Characteristics of the Study Population at Baseline.

	Overall	VFE at baseline	No VFE at baseline
Participants (n)	17	9	8
Age, years (mean ± SD)	62 ± 8	61 ± 6	62 ± 10
Gender, male/female (n)	11/6	6/3	5/3
Bamford classification, LACI/PACI/TACI (n)	6/8/3	4/4/1	2/4/2
Affected side, left/right (n)	11/6	6/3	5/3
Dominant hand, left/right (n)	2/15	2/7	0/8
Time between stroke and baseline measurement, days (mean ± SD)	15 ± 4	13 ± 4	16 ± 3
Clinical characteristics at baseline (median [IQR])			
NIHSS	7 [4.5-8]	5 [4.5-7.5]	7.5 [3.5-8.75]
FM-UE	21 [7.5-42.5]	42 [25.5-51]	7.5 [6.25-19]
FM-wrist	0 [0-3.5]	3 [1-6]	0 [0-0]
FM-hand	2 [0-6.5]	6 [2.5-10.5]	0 [0-1]
NC	1.95 [1.02-5.99]	1.95 [0.87-3.66]	2.89 [0.87-10.18]
EC	3.93 [3.36-4.77]	4.00 [2.94-4.91]	3.77 [3.30-4.80]
VC	0.20 [0.00-0.30]	0.15 [-0.10 to 0.27]	0.26 [0.18-0.42]

Abbreviations: VFE, voluntary finger extension; LACI, lacunar infarct; PACI, partial anterior circulation infarct; TACI, total anterior circulation infarct; NIHSS, National Institutes of Health Stroke Scale (range: 0-42 points); FM-UE, Fugl-Meyer upper extremity scale (range: 0-66 points); FM-wrist, Fugl-Meyer upper extremity scale, wrist subsection (range 0-10 points); FM-hand, Fugl-Meyer upper extremity scale, hand subsection (range 0-14 points); NC, velocity-dependent part of the neural component (N); EC, elastic component (N); VC, viscous component (N).

**Figure 3.**
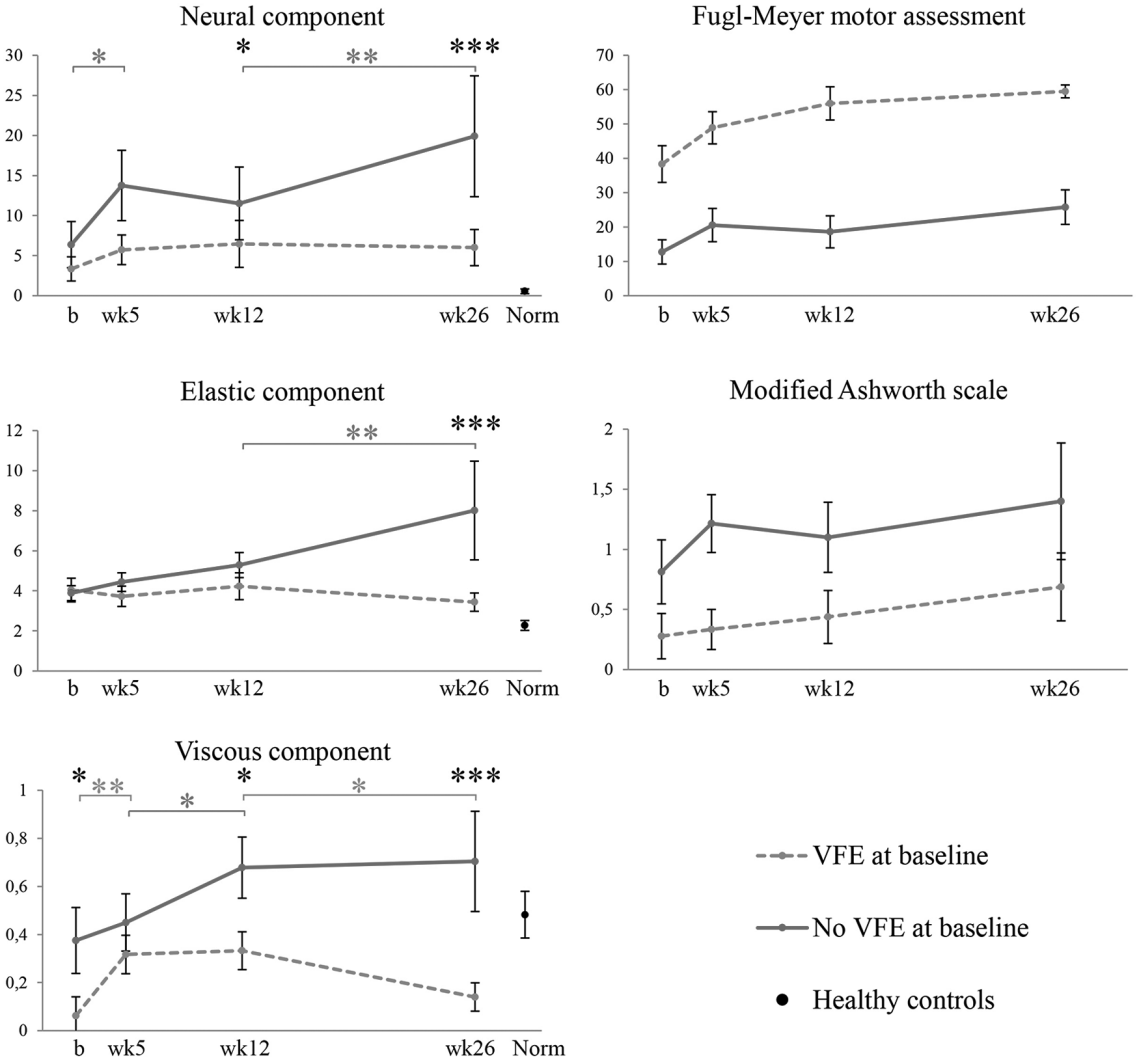
Time course of neural and biomechanical elastic and viscous components of wrist hyper-resistance, motor recovery, and total resistance to passive wrist extension post stroke. Values are mean (SE). b, baseline measurement within 3 weeks post stroke; wk, measurement week post stroke; VFE, voluntary finger extension. Baseline, n = 17 (VFE 9/no-VFE 8); week 5, n = 16 (VFE 9/no-VFE 7); week 12, n = 13 (VFE 8/no-VFE 5); week 26, n = 13 (VFE 8/no-VFE 5). *P < .05; **P < .01; ***P < .001; black *, significant difference between groups; green*/red*, significant change over time within the groups of patients with and without voluntary finger extension.

Figure 3 shows the averaged time course of the neural and biomechanical elastic and viscous components of wrist hyper-resistance, as well as the FM-UE and MAS scores. In patients with VFE at baseline, mean FM-UE scores improved from 38 points at baseline to 60 points (range 48-64) at week 26. In patients without VFE at baseline, mean FM-UE scores improved from 13 points at baseline to 26 points (range 13-42) at week 26. The mean total resistance to passive wrist extension, as manually measured with the MAS, increased in patients with VFE at baseline from 0.3 at baseline to 0.7 at week 26 and in patients without VFE at baseline from 0.8 at baseline to 1.4 at week 26. None of the patients received botulinum toxin injections during the study period.

### Time Course of Neural and Biomechanical Elastic and Viscous Components of Wrist Hyper-Resistance

Table 2 shows the results of the linear mixed model analyses for the total group, and for patients with and without VFE at baseline. For the total group, a significant increase in NC was found between baseline and week 5 (β = +4.04, *P* = .049). A significant increase in EC over time was found between baseline and week 26 (β = +1.37, *P* = .047), and a significant increase in VC was found between baseline and week 5 (β = +0.16, *P* = .028). The time course of the neural and biomechanical elastic and viscous components of wrist hyper-resistance between baseline and week 26 differed between the 2 stratified groups (Figure 3). In patients with VFE at baseline, no significant changes over time in components of wrist hyper-resistance were found, except for a significant increase in VC between baseline and week 5 (β = +0.25, *P* = .004), and a significant decrease of VC between weeks 12 and 26 (β = −0.19, *P* = .034). In patients without VFE at baseline, the NC showed a significant increase over time (β = +17.61, *P* < .001), with significant increases between baseline and week 5 (β = +5.55 N, *P* = .019), and between weeks 12 and 26 (β = +8.38 N, *P* = .004). EC showed a significant increase over time (β = +4.13, *P* < .001), with a significant increase between weeks 12 and 26 (β = +2.72 N, *P* = .005). VC significantly increased between weeks 5 and 12, and between baseline and week 26 (β = +0.22, *P* = .046, and β = +0.29, *P* = .008, respectively). The individual data of wrist hyper-resistance components over time are shown in Supplemental Table 1.

**Table 2.** Time Course of Neural and Biomechanical Elastic and Viscous Components of Wrist Hyper-Resistance in the First 6 Months Post stroke.^a^

	Baseline – week 5	Week 5 – week 12	Week 12 – week 26	Baseline – week 26
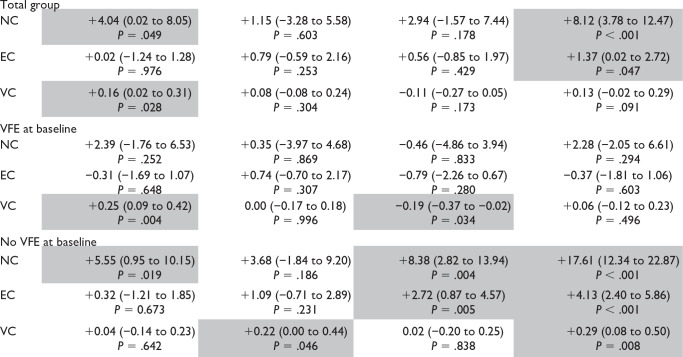				

Abbreviations: VFE, voluntary finger extension; NC, velocity-dependent part of the neural component (N); EC, elastic component (N); VC, viscous component (N).

a Values are estimated regression coefficients (β), 95% confidence interval, and probability estimates (*P*). Grey-filled boxes indicate significant values.

As shown in Table 3, the NC and VC were significantly higher at week 12 in patients without VFE at baseline, compared to patients with VFE at baseline (NC β = +9.52 N, *P* = .046, and VC β = +0.33 N, *P* = .029, respectively). The neural as well as both biomechanical components in patients without VFE at baseline were significantly higher at 26 weeks post stroke compared to those in patients with VFE at baseline (NC β = +18.36 N, *P* < .001, EC β = +4.35 N, *P* < .001, and VC β = +0.54 N, *P* < .001, respectively).

**Table 3.** Differences in Neural and Biomechanical Elastic and Viscous Components of Wrist Hyper-Resistance Between Patients With and Without Voluntary Finger Extension at Baseline in the First 6 Months Post stroke.^a^

	Baseline no-VFE vs VFE	Week 5 no-VFE vs VFE	Week 12 no-VFE vs VFE	Week 26 no-VFE vs VFE
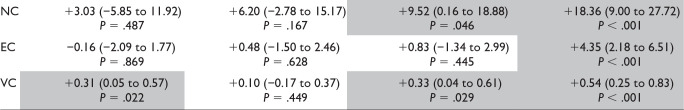				

Abbreviations: no-VFE, group of patients without voluntary finger extension at baseline; VFE, group of patients with voluntary finger extension at baseline; NC, velocity-dependent part of the neural component (N); EC, elastic component (N); VC, viscous component (N).

a Values are estimated regression coefficients (β), 95% confidence interval, and probability estimates (*P*). Grey-filled boxes indicate significant values.

At 26 weeks post stroke, a negative correlation coefficient was found between the FM-UE score and the NC (*r* = −0.54, *P* = .055) and between the FM-UE score and the EC (*R* = −0.73, *P* = .004) (Supplemental Figure 2).

### Healthy Reference Values

Healthy controls had a mean (SD) NC of 0.55 N (1.21), an EC of 2.27 N (1.01), and a VC of 0.48 N (0.40). Patients had significantly higher neural and elastic components, and a significantly lower viscous component at baseline compared with healthy controls (NC: *P* = .001; EC: *P* < .001; VC: *P* = .016). At 26 weeks post stroke, the NC in patients with VFE at baseline was still significantly higher than that in healthy controls (*P* = .010), while the EC in these patients was not significantly different from the reference value of healthy controls (*P* = .055). Patients with VFE at baseline had significantly lower VC values at 26 weeks post stroke (*P* = .027) compared with healthy controls, while the VC of the patients without VFE at baseline did not differ from the reference value of healthy controls (*P* = .649).

### Interaction Between Neural and Biomechanical Elastic and Viscous Components of Wrist Hyper-Resistance

As shown in Figure 3 for patients without VFE at baseline, the first significant increase in NC appeared in the time-window between baseline and week 5, preceding the first significant increase in EC between weeks 12 and 26. Correlation coefficients over time between the neural and biomechanical elastic and viscous components are shown in Supplemental Table 2. At baseline and week 5 post stroke, no significant association was found between the NC and the EC. From week 12 onward, the NC and the EC showed significant correlation coefficients, from 0.78 at week 12 to 0.80 at week 26.

## Discussion

The current prospective cohort study investigated the time course of wrist hyper-resistance in the first 6 months post stroke, separated into its neural and biomechanical elastic and viscous components using a commercially available measurement technique. First, as hypothesized, patients showing no VFE at baseline showed a gradual, significant increment in NC and subsequently also in the biomechanical EC and VC components of wrist hyper-resistance within the first 6 months post stroke, whereas no significant change in either of the components of wrist hyper-resistance over time was seen in patients showing VFE at baseline. Second, the main increase in NC in patients without VFE at baseline occurred within the first 5 weeks post stroke, paralleling the time-window of spontaneous motor recovery as reflected by FM-UE improvements.^32-34^ Last, our findings suggest that the increase in NC within the first 5 weeks post stroke in the group of patients without VFE preceded the increase in EC after 12 weeks post stroke.

The group of patients showing no VFE at baseline showed poor upper limb motor recovery in the first 6 months post stroke, as reflected by a FM-UE ≤42.^35^ As shown in previous studies, the absence of VFE at baseline is highly associated with absence of spontaneous neurobiological recovery and damage of the corticospinal tract (CST) early after stroke.^4,33^ The development of the neural component of wrist hyper-resistance early after stroke in patients with severe baseline motor deficits, as seen in our study, might be driven by enhanced multisynaptic descending pathways, when CST integrity is compromised^36-40^; however, this hypothesis requires further investigation. Moreover, the negative association between the FM-UE score and NC at week 26 suggests that the degree of spontaneous neurobiological recovery is associated with a decrease in the severity of wrist hyper-resistance.

In this study with a relatively small sample size, we used the presence of VFE at baseline as a proxy for CST intactness allowing to dichotomize the study population into a group of patients with poor and with good upper limb motor recovery post stroke.^1,41^ In addition, other markers of CST integrity such as transcranial magnetic stimulation (TMS)–induced motor-evoked potentials (MEPs) of the extensor carpi radialis^33^ or the adductor digiti minimi,^42^ diffusion tensor imaging (DTI) fractional anisotropy,^43^ weighted CST lesion load (wCST-LL) in magnetic resonance imaging^44^ as well as kinetic and kinematic performance assays of behavioral restitution^45^ might improve the accuracy in identifying those patients that will develop wrist hyper-resistance early post stroke and might strengthen evidence on the relationship of wrist hyper-resistance components with upper limb motor recovery, its timeline and underlying pathophysiological concepts. However, these studies require larger sample sizes than presently available.

In our opinion, the observed changes in the neural component reflect neural repair processes early after stroke, further influencing behavioral restitution, as measured with the FM-UE. The absence of neural and biomechanical components of wrist hyper-resistance are conditional for optimal behavioral restitution. Our findings are consistent with a previous study,^36^ which suggested that the velocity-dependent increase in muscle tone after damage to the CST due to stroke can be explained by enhanced multisynaptic, reticulospinal pathways in patients with chronic stroke. Confirmation of the enhancement of different multisynaptic pathways using neuroimaging or neurophysiological techniques, such as TMS-MEPs and DTI fractional anisotropy, and its role in the development in wrist hyper-resistance requires further investigation.

Interestingly, the NC further increased after the time-window of spontaneous motor recovery, between weeks 12 and 26, which implies that the development of the NC is not influenced by motor recovery alone. This increase of NC may result from biomechanical tissue property alterations, as shown by the high correlation between NC and EC from 12 weeks onward. Besides the increase in velocity-dependent wrist hyper-resistance, as represented by the NC, pathological neuromuscular activation may also comprise increased involuntary background activation. This involuntary background activation, which is measured by resting torque by the NeuroFlexor, may also cause an increase in the elastic component.^46^

Our findings of increased neural and biomechanical components of wrist hyper-resistance in patients with poor motor recovery are in line with the results of a previous study,^15^ using a haptic robot device with a validated EMG-driven wrist model in 36 patients in the first 6 months post stroke. The results of both the present and aforementioned study^15^ suggest that components of wrist hyper-resistance show large interindividual variability, which suggests that the level of motor recovery as well as additional factors, such as genetic factors, lesion location, and premorbid muscle morphology, play a role.

As the NC mainly increases within the time-window of spontaneous neurobiological recovery, it is of interest to know whether the development of the NC restricts motor recovery, and if early reduction of the NC, for example using botulinum toxin, would positively influence motor recovery post stroke. The influence of the development of NC on motor recovery, and the effect of early reduction by botulinum toxin, should be further investigated.

As expected, our group of patients with good motor recovery (ie, those presenting with VFE at baseline), showed no change in wrist hyper-resistance over time. However, against our expectations, the NC in this group 6 months after stroke onset was significantly higher than the values of healthy controls, whereas the EC approached reference values within 6 months post stroke. These data suggest that some degree of CST intactness, represented by the presence of VFE at baseline, is needed for motor recovery, apparently without interference from a slightly increased NC. In contrast to the NC and EC, the VC in the patients with poor motor recovery showed equivalent values to the healthy controls, whereas it showed decreased values compared with healthy controls in patients with good motor recovery. It should be noted that VC has hardly been investigated early post stroke, and in our study, it contributed only 3% to the total wrist hyper-resistance measured with the NeuroFlexor. De Vlugt et al^47^ found comparable results with higher viscosity in the ankle in patients after stroke with higher Ashworth scale values. The decreased VC values in patients with VFE at baseline compared with healthy reference values might result from antagonistic muscle tension, problems of the biomechanical model handling these data, or lack of responsiveness.^23^

The NeuroFlexor model includes 4 slow and 9 fast wrist extension movements in the analysis of the components of wrist hyper-resistance. Analysis of the separate fast movements of one measurement session for all patients at baseline revealed a significant reduction of 17% in the NC between the first and last fast movement (paired *t*-test, mean difference −0.95 N, 95% confidence interval −1.81 to −0.10 N, *P* = .031) (Supplemental Figure 3). This reduced resistance over repeated movements may be due to, for example, an effect of time-dependent viscosity,^48,49^ varying background activation over time or mechanical hysteresis. To handle these still unknown nonlinear effects, it is important to use standardized measurement protocols with a detailed description of the fixed number of repeated movements, the position and instruction of the participants, and extensive training for assessors. Moreover, the underlying mechanisms that contribute to the nonlinear behavior of resistance to passive movement after stroke need further investigation.^50^

### Study Limitations

It should be noted that our study included only a small number of subjects. Nevertheless, the findings were robust enough to show significant changes in components of wrist hyper-resistance over time and significant differences between 2 subgroups of patients. Being sensitive to outliers in this small sample, nonparametric statistics led to the same conclusions when compared with current linear mixed model analyses. Furthermore, due to the different components of wrist hyper-resistance tested in this explorative study, we are also aware of multiple comparisons applied, suggesting that replication of current findings in a larger sample is needed.

Furthermore, the NC in both groups at baseline was already increased compared with healthy controls. With that, the exact moment of onset remains unclear in absence of measurements applied in the first days after stroke onset.^32^ Further research in a larger population with more and earlier started measurements serially applied at fixed time-points within the first 12 weeks post stroke is needed to provide independent confirmation of our findings. Finally, this study only included patients with ischemic stroke, a generalization of study results to patients with hemorrhagic stroke should therefore be cautioned.

In recent years, several instrumented measurement techniques have been developed to quantify neural and biomechanical components of hyper-resistance, which differ in complexity and modelling method.^51-55^ In the absence of an appropriate gold standard, no single most valid method can be identified. Being interested in serially applied, within-subjects’ measurements, we used the commercially available and portable NeuroFlexor method to quantify neural and biomechanical components of wrist hyper-resistance in a clinical setting. However, the biomechanical model used for discriminating between neural and biomechanical components of hyper-resistance has some limitations. First, the underlying biomechanical model assumes linearity, however, this approach does not address the nonlinear features as length and velocity-dependent threshold of the stretch reflex,^56^ and the velocity-dependent VC. Second, it should be noted that the wrist was extended at 2 arbitrarily selected velocities (5 and 236 deg/s, respectively), which are assumed to be below and above expected reflex threshold velocities, respectively.^47^ Third, the wrist and finger flexor muscles were extended over a fixed 50° range around the neutral position of the wrist, regardless of the individual’s passive range of motion. The device might therefore be insensitive to small changes in EC, as well as early muscle shortening.^57^ Fourth, since it does not measure EMG but only resting torque of the hand before stretch onset, the NeuroFlexor is not able to specifically control and correct for the influence of increased involuntary background activation on wrist hyper-resistance.^46^ Fifth, this involuntary background activation, that is, the non-velocity-dependent part of neural activity, may also manifest in the non-velocity, length-dependent component of the total wrist joint resistance which is assumed to reflect the biomechanical EC component according to the underlying biomechanical model of the NeuroFlexor.^22^ Finally, the NeuroFlexor protocol requires a minimal passive wrist extension of 40°. In our study, 1 patient with poor motor recovery developed restriction of the passive wrist range of motion with extended fingers to less than 40°, and was therefore unable to comply with the protocol and could not be followed longitudinally from 12 weeks onward.

### Future Research

The present findings require further replication and validation in a larger population adopting a multimodal approach to better understand the mechanisms underlying the increase in neural and biomechanical components of wrist hyper-resistance in patients with poor upper limb motor recovery. This knowledge about the underlying mechanisms might improve our understanding in the distinction between neural repair processes and its interaction with behavioral restitution in recovery of quality of movement early after stroke.^45^ Second, further investigation is needed into the role of different multisynaptic pathways, such as the reticulospinal tract, in the development of spasticity, for instance using acoustic startle reflexes (ie, StartReact phenomenon).^58^ Third, further research is needed to investigate other predisposing between-subject factors explaining the heterogeneity between subjects in the development of wrist hyper-resistance post stroke, next to severity of upper limb paresis alone, such as genetic factors, lesion location and premorbid muscle morphology. Additionally, further refinement of the quantification of the neural component of wrist hyper-resistance, using EMG activity, is needed to differentiate between an increase in velocity-dependent spasticity and non-velocity-dependent involuntary background activation,^59^ and to reveal more about the moment of change. Last, the quantification of neural and biomechanical components of wrist hyper-resistance, including the velocity-dependent VC that did contribute less than 3% of the total wrist resistance, requires further validation of the NeuroFlexor method with more sophisticated system identification techniques in the next future.

## Supplemental Material

Supplemental_material_v3 – Supplemental material for Time Course of Wrist Hyper-Resistance in Relation to Upper Limb Motor Recovery Early Post stroke Click here for additional data file. Supplemental material, Supplemental_material_v3 for Time Course of Wrist Hyper-Resistance in Relation to Upper Limb Motor Recovery Early Post stroke by Aukje Andringa, Carel Meskers, Ingrid van de Port, Erwin van Wegen and Gert Kwakkel in Neurorehabilitation and Neural Repair
