# Supplementary material for: Time Course of Wrist Hyper-Resistance in Relation to Upper Limb Motor Recovery Early Post Stroke
Source: Neurorehabil Neural Repair. 2020 Jun 7;34(8):690–701. doi: 10.1177/1545968320932135 (PMC7502985; doi:10.1177/1545968320932135)
Supplement: Supplemental_material_v3 – Supplemental material for Time Course of Wrist Hyper-Resistance in Relation to Upper Limb Motor Recovery Early Post stroke [file Supplemental_material_v3.docx]

***Supplemental material***

***Supplemental File 1.*** Biomechanical model of the NeuroFlexor

In the biomechanical model of the NeuroFlexor, previously described by Lindberg et al^1^, the total measured resisting force (F_m_) during passive wrist extension is a summation of passive elastic force (F_p_), viscous force (F_v_), reflexive force (F_r_), and inertial forces of the limb and the moving parts of the device (F_in_), described as:

F_m_(θ) = F_p_(θ) + F_v_(θ) + F_r_(θ) + F_in_(θ), where θ denotes a specific angle.

In the model, four force points in the resistance trace of the slow and fast displacements are used to estimate the different components of the total measured passive force. P0 is the resting torque of the hand before onset of stretch. Two force points are defined within the fast passive wrist extension movement (236°/s): P1, the initial peak in resistance, and P2, the late peak in resistance (Figure B). One force point (P3) is defined at the end position of the slow wrist extension movement (5 °/s) (Figure A). Resting torque (P0) is subtracted from P1, P2 and P3 prior to further calculations. Two slow and two fast movements without the hand and forearm fastened to the device are run as a reference for the mechanical resistance by the hand platform on the force sensor.

The **inertia component (IC)** corresponds to the force resisting the acceleration of the hand and is calculated in the model as:

IC = *m* × *a*,

where *m* is the mass of the hand and the movable platform, and *a* is the angular acceleration (21 m/s^2^). The mass of the hand is estimated to be 0.6% of the total body weight.

The **elastic component (EC)** is a length-dependent resisting force which increases when the muscles are stretched, with an exponential increase when the muscle is stretched close to its end range. The EC is recorded 1 second after the end of the slow movement. The EC corresponds to P3, i.e. the fully stretched position during the slow movement (Figure A).

The **viscous component (VC)** is produced by the sliding muscle fibers, and is velocity-dependent. Lindberg et al^1^ assumed that the viscous resistance is highest during the initial acceleration and continues at a lower level during further extension movement. To calculate the viscous component, first, the early viscosity component (VC_P1_) is calculated.

VC_P1_ = Total force_P1_ − IC,

where Total force_P1_ is the measured force at P1 (Figure B), and IC the inertial component calculated as above. Since there is a comparatively stable relationship between the early and late viscosity, Lindberg et al^1^ assumed that the late viscosity is approximately 20% of the early viscosity.

VC = VC_P1_ × 0.2.

Finally, P2 is defined as the late force peak during the fast wrist extension movement (Figure B) and consists of the neural, viscous and elastic component together. **The velocity-dependent part of the neural component (NC)** is estimated by:

NC = Total force_P2_ − (EC + VC).


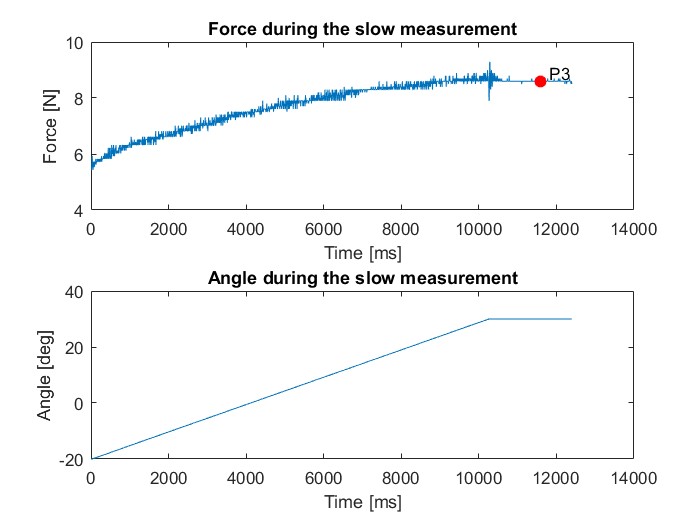


P0

A. Slow movement


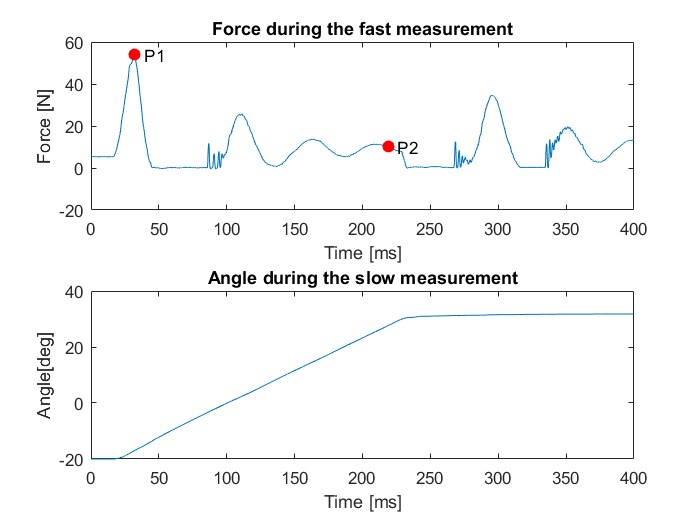


P0

B. Fast movement

**References**

1. Lindberg PG, Gaverth J, Islam M, Fagergren A, Borg J, Forssberg H. Validation of a new biomechanical model to measure muscle tone in spastic muscles. *Neurorehabil Neural Repair*. 2011;25(7):617-25.


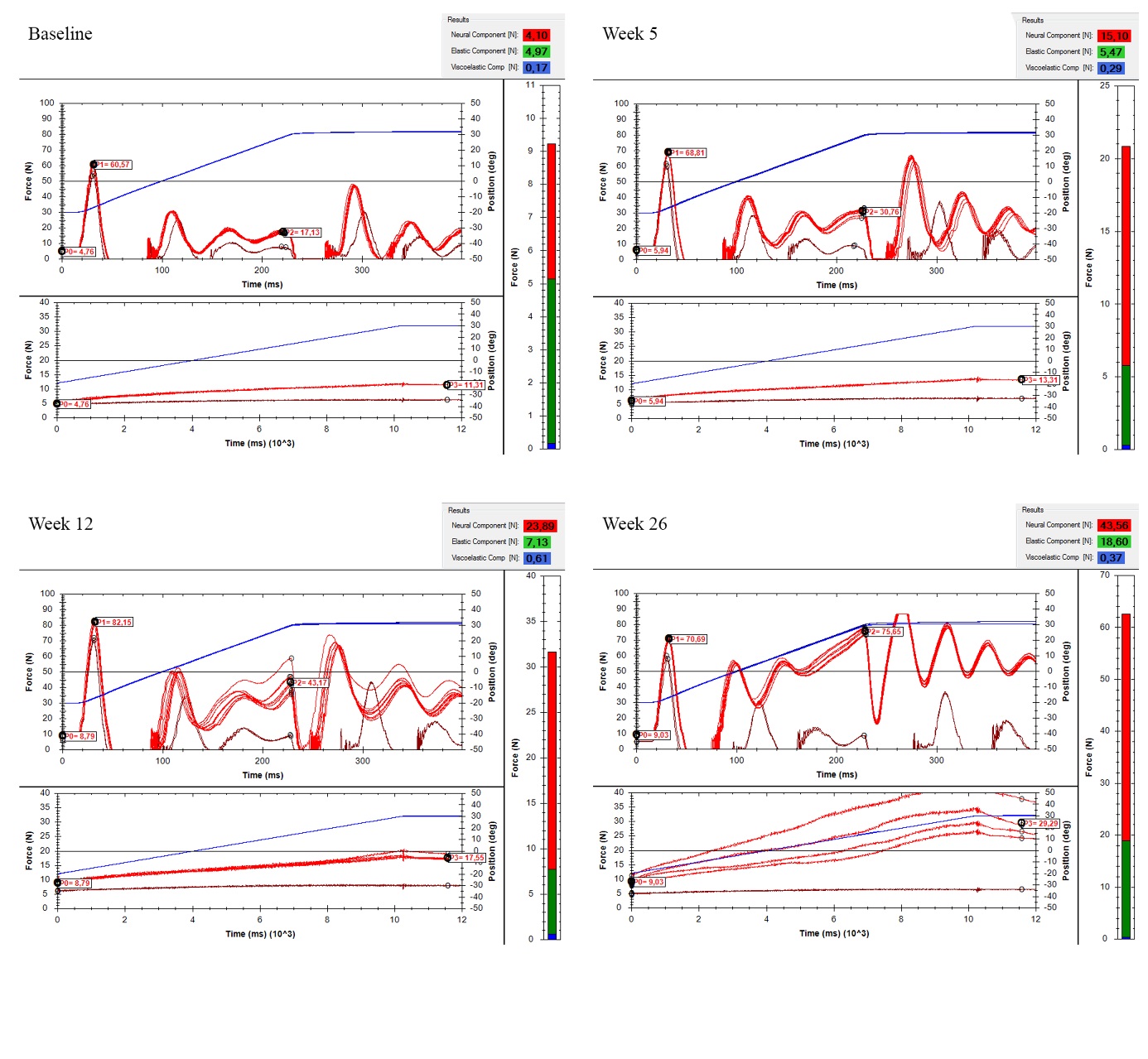
***Supplemental Figure 1a.*** Raw NeuroFlexor data over time of a patient showing no voluntary finger extension at baseline (patient 7). Red lines represent resistance traces of nine fast and four slow movements; blue lines represent wrist angle during the movements.


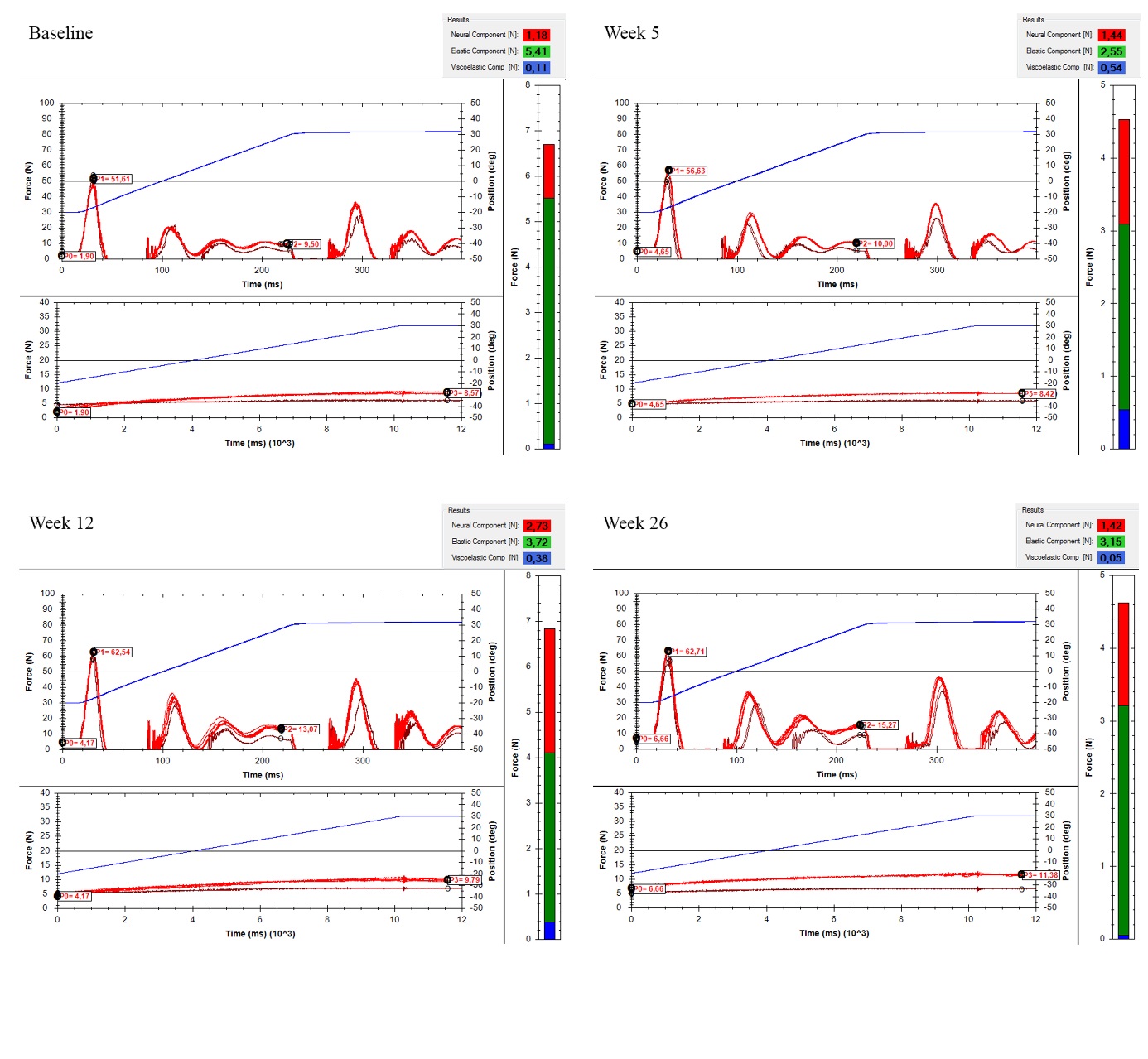


***Supplemental Figure 1b.*** Raw NeuroFlexor data over time of a patient showing voluntary finger extension at baseline (patient 4). Red lines represent resistance traces of nine fast and four slow movements; blue lines represent wrist angle during the movements.


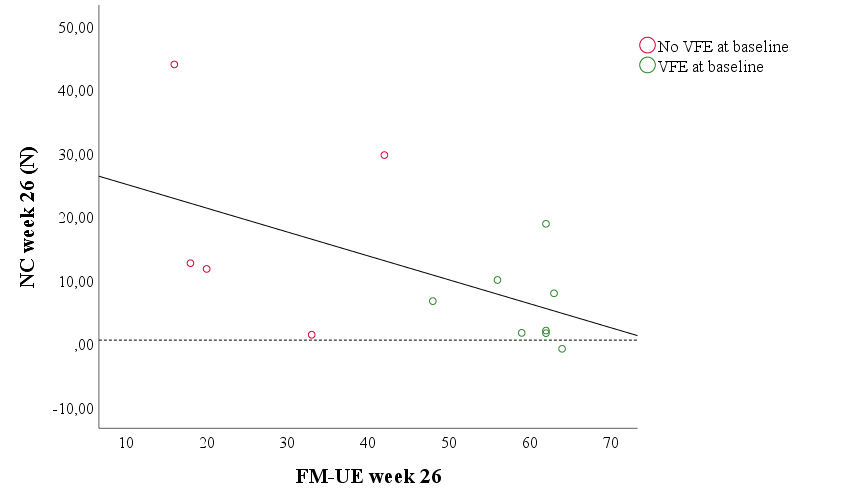


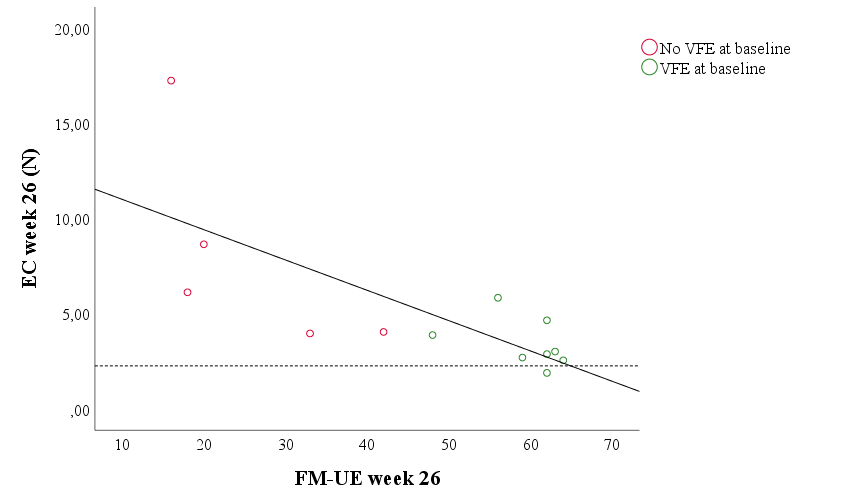


***Supplemental Figure 2.*** Association between the Fugl-Meyer upper extremity scale and components of wrist hyper-resistance at 26 weeks post stroke

FM-UE: Fugl-Meyer upper extremity scale; NC: velocity-dependent part of the neural component; EC: elastic component; dotted line: healthy reference value.


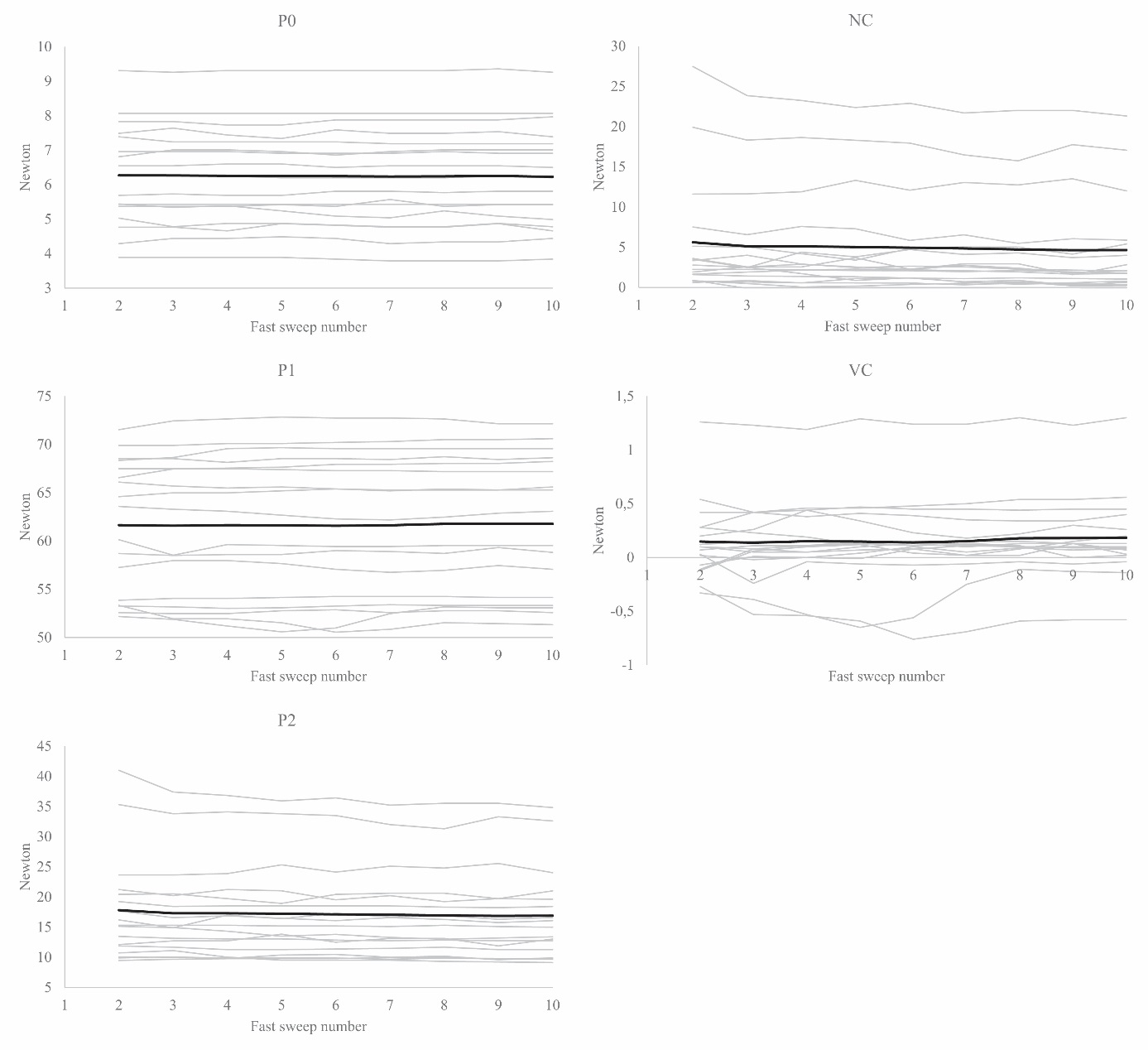
***Supplemental Figure 3***. Individual and mean results of nine repetitive fast movements of a NeuroFlexor measurement session at baseline for resting torque (P0), initial resistance peak (P1), late resistance peak (P2), velocity-dependent part of the neural component (NC), and viscous component (VC). Each grey line represents one subject and the black line represents the mean value. Note that the elastic component is not affected by the resistance force of the fast movements.

***Supplemental Table 1.*** Individual data of wrist hyper-resistance components over time

|  |  | NC | | | | EC | | | | VC | | | | FM-UE | | | | MAS | | | |
| --- | --- | --- | --- | --- | --- | --- | --- | --- | --- | --- | --- | --- | --- | --- | --- | --- | --- | --- | --- | --- | --- |
|  | VFE | b | w5 | w12 | w26 | b | w5 | w12 | w26 | b | w5 | w12 | w26 | b | w5 | w12 | w26 | b | w5 | w12 | w26 |
| 1 | 0 | 1.32 |  | 0.64 | 1.39 | 1.98 |  | 4.46 | 3.97 | 0.18 |  | 0.44 | 0.24 | 6 |  | 13 | 33 | 0 |  | 0 | 0 |
| 2 | 1 | 2.32 | 6.46 | 3.49 | 10.01 | 4.07 | 3.26 | 4.36 | 5.85 | 0.15 | 0.38 | 0.27 | 0.19 | 43 | 49 | 54 | 56 | 0 | 0 | 0 | 2 |
| 3 | 1 | 14.86 | 18.38 | 25.81 | 18.86 | 5.45 | 5.55 | 7.98 | 4.66 | 0.31 | 0.78 | 0.85 | -0.12 | 40 | 56 | 63 | 62 | 1+ | 1 | 1 | 1 |
| 4 | 1 | 1.57 | 1.57 | 2.91 | 1.69 | 4.36 | 2.54 | 3.78 | 2.71 | -0.12 | 0.56 | 0.28 | 0.28 | 50 | 55 | 60 | 59 | 0 | 0 | 0 | 0 |
| 5 | 1 | 4.78 | 3.10 | 1.73 | 1.63 | 4.00 | 4.26 | 5.32 | 2.89 | 0.15 | 0.23 | 0.20 | -0.10 | 42 | 51 | 63 | 62 | 0 | 0 | 0 | 0 |
| 6 | 1 | 0.31 | 1.67 |  |  | 7.48 | 6.28 |  |  | -0.07 | 0.10 |  |  | 18 | 34 |  |  | 0 | 1 |  |  |
| 7 | 0 | 4.00 | 13.38 | 23.20 | 43.98 | 5.28 | 6.05 | 6.19 | 17.25 | 0.05 | 0.22 | 0.54 | 0.37 | 6 | 14 | 14 | 16 | 1+ | 1+ | 1+ | 3 |
| 8 | 0 | 11.17 | 31.55 |  |  | 3.25 | 4.94 |  |  | 0.22 | 0.24 |  |  | 7 | 13 | 13 | 13 | 2 | 2 | 3 | 3 |
| 9 | 0 | 24.35 | 28.03 |  |  | 3.90 | 3.59 |  |  | 0.30 | 0.18 |  |  | 34 | 41 |  |  | 1 | 1 |  |  |
| 10 | 1 | 0.33 | 1.73 | 4.95 | 2.05 | 2.34 | 1.97 | 1.73 | 1.90 | 0.22 | 0.13 | 0.11 | 0.22 | 52 | 60 | 60 | 62 | 0 | 0 | 0 | 0 |
| 11 | 1 | 2.54 | 8.08 | 6.14 | 6.69 | 3.93 | 4.05 | 4.18 | 3.89 | 0.35 | 0.38 | 0.26 | 0.34 | 9 | 18 | 23 | 48 | 1 | 1 | 1 | 1 |
| 12 | 1 | 1.42 | 8.73 | 7.86 | 7.92 | 3.54 | 3.79 | 4.14 | 3.02 | -0.05 | 0.27 | 0.35 | 0.16 | 33 | 60 | 64 | 63 | 0 | 0 | 1+ | 1+ |
| 13 | 0 | 0.42 | 3.04 | 10.72 | 11.76 | 4.72 | 3.95 | 6.48 | 8.65 | 0.46 | 0.59 | 1.06 | 1.00 | 8 | 10 | 17 | 20 | 0 | 0 | 1 | 1 |
| 14 | 1 | 1.95 | 1.84 | -1.17 | -0.83 | 1.19 | 1.84 | 2.34 | 2.56 | -0.38 | 0.02 | 0.34 | 0.15 | 58 | 57 | 61 | 64 | 0 | 0 | 0 | 0 |
| 15 | 0 | 0.72 | 4.19 | 2.68 | 12.67 | 3.48 | 2.34 | 3.22 | 6.13 | 0.20 | 0.27 | 0.45 | 0.55 | 7 | 7 | 12 | 18 | 0 | 1 | 1+ | 1+ |
| 16 | 0 | 1.79 | 5.38 |  |  | 3.64 | 5.00 |  |  | 0.30 | 0.61 |  |  | 13 | 32 |  |  | 1 | 1+ |  |  |
| 17 | 0 | 7.21 | 10.72 | 20.35 | 29.70 | 4.83 | 5.18 | 6.08 | 4.05 | 1.29 | 1.04 | 0.90 | 1.36 | 21 | 27 | 37 | 42 | 1 | 1+ | 1+ | 1+ |

VFE: voluntary finger extension (1: VFE at baseline; 0: no VFE at baseline); NC: velocity-dependent part of the neural component (N); EC: elastic component (N); VC: viscous component (N); FM-UE: Fugl-Meyer upper extremity scale; MAS: modified Ashworth score wrist and finger flexor muscles.

***Supplemental Table 2.*** Pearson correlation coefficients for neural and biomechanical elastic and viscous components of wrist hyper-resistance in the first 6 months post stroke

|  | NCb | NC5 | NC12 | NC26 | ECb | EC5 | EC12 | EC26 | VCb | VC5 | VC12 | VC26 |
| --- | --- | --- | --- | --- | --- | --- | --- | --- | --- | --- | --- | --- |
| NCb | 1 | **0.85**  **.000** | 0.73  .005 |  |  |  | 0.72  .006 |  |  |  |  |  |
| NC5 | - | 1 | **0.88**  **.000** | 0.72  .009 |  |  | 0.73  .007 |  |  |  |  |  |
| NC12 | - | - | 1 | **0.85**  **.000** | **0.76**  **.003** | **0.87**  **.000** | **0.78**  **.002** |  |  | 0.61  .034 | 0.67  .012 |  |
| NC26 | - | - | - | 1 | 0.68  .011 | **0.79**  **.002** | 0.60  .029 | **0.80**  **.001** |  |  |  |  |
| ECb | - | - | - | - | 1 | **0.77**  **.000** | **0.81**  **.001** |  |  |  |  |  |
| EC5 | - | - | - | - | - | 1 | **0.89**  **.000** |  |  |  |  |  |
| EC12 | - | - | - | - | - | - | 1 |  |  | 0.68  .016 | **0.76**  **.003** |  |
| EC26 | - | - | - | - | - | - | - | 1 |  |  |  |  |
| VCb | - | - | - | - | - | - | - | - | 1 | 0.74  .001 | 0.59  .035 | **0.75**  **.004** |
| VC5 | - | - | - | - | - | - | - | - | - | 1 | 0.72  .008 |  |
| VC12 | - | - | - | - | - | - | - | - | - | - | 1 | 0.63  .022 |
| VC26 | - | - | - | - | - | - | - | - | - | - | - | 1 |

Values are Pearson correlation coefficients with p-values. Only significant correlation coefficients are shown. In bold good to excellent associations (*r* > 0.75). NC: velocity-dependent part of the neural component; EC: elastic component; VC: viscous component.
